# Supplementary material for: Complexome profiling on the Chlamydomonas lpa2 mutant reveals insights into PSII biogenesis and new PSII associated proteins
Source: J Exp Bot. 2021 Aug 26;73(1):245–62. doi: 10.1093/jxb/erab390 (PMC8730698; doi:10.1093/jxb/erab390)
Supplement: erab390_suppl_Supplementary_Dataset_S1 [file erab390_suppl_supplementary_dataset_s1.zip › Supplemental Dataset 1 - Excel List and all profiles/readMe.docx]

# ProteinDistances Table

**Abbreviations**

- WT	wild type
- Mut	*lpa2* knock out mutant
- Sample	6 samples were loaded to the gel; 3x WT and 3x Mut; One sample consists of 36 slices
- Slice	A sample gel lane was subdivided into 36 slices

The Excel spread sheet contains several additional information about every measured protein. **The name in the first column directly links to detailed charts for the respective protein.** *Note: Please save the directory to your local machine and allow Excel to open external content.*

Intensities of wild type (WT) and *lpa2* (Mut) are normed as follows:

- The absolute intensity sum of every protein and gel slice was calculated for each of the six samples (3xWT and 3xMut).
- The mean of the six total intensity sums was calculated (TImean).
- To determine the normalized intensities a correction factor for each sample was calculated:
  - The total intensity sum of each sample was divided by TImean to get the correction factor (CF_i_), resulting in CF_WT1_, CF_WT2_, CF_WT3_, CF_Mut1_, CF_Mut2_, and CF_Mut3_.
- Every intensity value was divided by the respective correction factor, to adjust the equalized sample sums.

For further analysis, proteins identified by non-proteotypic peptides were discarded. A Welch test was performed for each protein by taking the sums of all 36 slice intensities for each WT and Mut.

**Column A:** Cre Identifier based on JGI genome release version 5.5

**Column B**: Trivial name

**Column C:** MapMan Annotation Description

**Column D:** MapMan Annotation Term

**Column E:** Gene Ontology Annotation

**Column F:** Subcellular localization

**Column G:** Number of nonzero replicate signals (WT)

**Column H:** Number of nonzero replicate signals (Mut)

**Column I:** p value of Welch test on intensity sums (WT_1-3_ vs Mut_1-3_)

**Column J:** Euclidean distance adjusted by the maximal mean intensity of WT means or Mut means (see Column L).

**Note:** Distance is 36, if there was no signal in WT or Mut respectively!

**Column K:** Euclidean distance of intensity mean signals.

**Column L:** Maximum of (mean of 36 mean intensities (WT)) and (mean of 36 mean intensities (Mut))

**Column M/N:** Slice indices of local maxima within the signal (Peak)

**Note:** A peak was identified when:

1. ${intensity}_{s-1}< {intensity}_{s}>{intensity}_{s+1}$
2. ${intensity}_{s}>({maxIntensity}_{p}\cdot0.1)$

where: $s=slice index, p=protein of interest$

**Columns O-AX:** mean intensities of three WT samples

**Columns AY-CH:** mean intensities of three Mut samples

# ProteinDistances chart

The ProteinDistances.html contains all protein mean intensity signals for WT and Mut. If no protein was detectable, the respective lane is transparent. For better comparability the signals were zTransformed. On the right panel the adjusted euclidean distance is given.

Note1: If a signal is completely missing in either WT or Mut, the distance is 36.
Note2: The more replicates showed a signal for a protein, the lower the distance tends to be.

The chart is interactive. Click and pan vertically to zoom into details.

# Protein charts

In /plots overview charts for every protein is given as ‘[TrivialName]_[CreIdentifier]'. If no trivial name is associated, the file starts with '_'.

**Protein charts are ordered as follows:**

- 1/3: raw data
- 2/3: normed data
- 3/3: intensity means with standard deviation
- Chart descriptions contain distance metrics, MapMan annotation, and pValues of a Welch test regarding the intensity sums
